# Stabilization of HIF-1α in Human Retinal Endothelial Cells Modulates Expression of miRNAs and Proangiogenic Growth Factors

**DOI:** 10.3389/fphar.2020.01063

**Published:** 2020-07-17

**Authors:** Francesca Lazzara, Maria Consiglia Trotta, Chiara Bianca Maria Platania, Michele D’Amico, Francesco Petrillo, Marilena Galdiero, Carlo Gesualdo, Settimio Rossi, Filippo Drago, Claudio Bucolo

**Affiliations:** ^1^ Department of Biomedical and Biotechnological Sciences, School of Medicine, University of Catania, Catania, Italy; ^2^ Department of Experimental Medicine, Division of Pharmacology, University of Campania “Luigi Vanvitelli”, Naples, Italy; ^3^ Eye Clinic, Multidisciplinary Department of Medical, Surgical and Dental Sciences, University of Campania “Luigi Vanvitelli”, Naples, Italy; ^4^ Center for Research in Ocular Pharmacology-CERFO, University of Catania, Catania, Italy

**Keywords:** hypoxia-inducible-factor-1α, vascular endothelial growth factor, transforming growth factor beta, retina, diabetic retinopathy, inflammation

## Abstract

Retinal hypoxia is one of the causative factors of diabetic retinopathy and is also one of the triggers of VEGF release. We hypothesized that specific dysregulated miRNAs in diabetic retinopathy could be linked to hypoxia-induced damage in human retinal endothelial cells (HRECs). We investigated in HRECs the effects of chemical (CoCl_2_) hypoxia on the expression of HIF-1α, VEGF, PlGF, and of a focused set of miRNAs. We found that miR-20a-5p, miR-20b-5p, miR-27a-3p, miR-27b-3p, miR-206-3p, miR-381-3p correlated also with expression of TGFβ signaling pathway genes in HRECs, challenged with chemical hypoxic stimuli. In conclusion, our data suggest that retinal angiogenesis would be promoted, at least under HIF-1α activation, by upregulation of PlGF and other factors such as miRNAs, VEGFA, and TGFβ1.

## Introduction

Diabetic retinopathy (DR), a complication of diabetes, is a microvascular disease with a strong inflammatory imprinting. Vascular endothelial growth factor (VEGF) is a key player in retinal neovascularization, and intraocular injections of anti-VEGF agents are currently the established therapies for diabetic macular edema, along with steroids (Bandello et al., 2012). Although not fully elucidated, alterations in retinal hemodynamics and reduced blood flow may be detrimental for DR, along with uncontrolled hyperglycemia (Schmetterer and Wolzt, 1999; Schmidl et al., 2015). Furthermore, during DR progression, local or global changes in retinal oxygenation may cause the development of hypoxic areas (Arden and Sivaprasad, 2012) and oxidative stress (Bucolo et al., 2006). Similar to the etiopathogenesis of retinopathy of prematurity (ROP), induction of hypoxia-inducible factor-1 α (HIF-1α) may be responsible for the production of vascular endothelial growth factor (VEGFA), which is the main cause of retinal neovascularization (Aiello et al., 1994; Arjamaa and Nikinmaa, 2006; Abu El-Asrar et al., 2012). Furthermore, HIF-1α and VEGFA crosstalk in ocular neovascularization has been widely investigated (Ozaki et al., 1999; Rodrigues et al., 2016). In particular, the HIF-1α inhibition strategy has also been explored for treatment of retinal neovascularization (Iwase et al., 2013; D’Amico et al., 2015; D’Amico et al., 2017; Zeng et al., 2017).

Besides VEGFA, HIF-1α can also induce the placental growth factor (PlGF) (Zimna and Kurpisz, 2015; Charnock-Jones, 2016; Mitsui et al., 2018), an emerging target in retinal neovascular diseases (Kwon and Jee, 2018; Lee et al., 2018; Saddala et al., 2018; Lazzara et al., 2019; Van Bergen et al., 2019). Furthermore, HIF-1α is involved in expression of several microRNAs (miRNAs), that are named HypoxamiRs if they bear in their promoter region the hypoxia responsive elements (HREs) (Nallamshetty et al., 2013; Bertero et al., 2017). Indeed, HypoxamiRs, regulated by HIF-1α dependent or independent mechanisms, are tightly involved in molecular and cellular changes triggered by hypoxia (Cottrill et al., 2014; Gee et al., 2014; Greco et al., 2014; Bertero et al., 2017). Moreover, several genes, that are target of HypoxamiRs, belong to the VEGFR2 signaling pathway (Gupta et al., 2018). This pathway regulates angiogenic response of endothelial cells (Abhinand et al., 2016), and represents the target of current approved treatments for neovascular retinal degenerations (Bandello et al., 2012). We recently evidenced the dysregulation of expression pattern of 8 miRNAs (miR-20a-5p, miR-20a3p, miR-20b-5p, miR-106a-5p, miR-27a-5p, miR-27b-3p, miR-206-3p, and miR-381-3p) in retina and serum of diabetic mice, representing intriguing and potent mediators in the DR pathological mechanisms (Platania et al., 2019). HREs were found in promoter region of miR-20a, miR-20b, miR-106, miR-27a, that indeed, have been enlisted as Hypoxamirs (Nallamshetty et al., 2013). Although HREs are not present in miR-206-3p, miR-381 and miR-27b promoter regions, these miRNAs were found to be modulated in several hypoxic experimental setting (Yue et al., 2013; Choudhry and Mole, 2016; Gupta et al., 2018; Lu et al., 2018).

Therefore, we hereby hypothesized that these eight miRNAs could also be involved in activation of HIF-1/angiogenic axis in retinal endothelial cells. With this aim, we stabilized, by cobalt chloride treatment, HIF-1α protein in human retinal endothelial cells (HRECs), in order to analyze the activation of HIF-1/VEGFA-PlGF axis, along with expression of a focused set of miRNAs, previously found to be dysregulated in an *in vivo* model of DR (Platania et al., 2019). A bioinformatic approach guided the identification and *in vitro* validation of alternative target genes of miRNAs, dysregulated after inhibition of HIF-1α degradation. We analyzed the expression of genes of the TGFβ (Transforming growth factor beta) signaling pathway, which is an emerging target in DR (Li et al., 2018; Stafiej et al., 2018) and was found to be one of top pathways modulated by HypoxamiRs target genes (Gupta et al., 2018).

## Material and Methods

### Reagents

Mouse monoclonal anti-HIF-1α (catalog n. sc-13515), mouse anti-GAPDH (catalog n. 2118) antibodies were purchased from Santa Cruz Biotechnology, Inc. (CA, USA), and Cell-Signaling Technology (Leiden, Netherlands), respectively. Secondary goat anti-mouse IRDye 680LT, (catalog n. 926-68020) were purchased from LI-COR (Lincoln, NE, USA). Cobalt chloride (0.1 M solution, catalog n. 15862) from Sigma-Aldrich (Saint Louis, MO, USA).

### Cell Culture

Human retinal endothelial cells were purchased from Innoprot^®^ (Derio – Bizkaia, Spain). Cells were cultured at 37°C, in humidified atmosphere (5% CO_2_), in Endothelial cell medium (ECM) supplemented with 5% fetal bovine serum (FBS), 1% ECGS (Endothelial Cell Growth Supplement) and 100 U/ml penicillin 100 μg/ml streptomycin. HRECs (cell passage number 4) for each experiment were seeded setting 4×10^5^ as final cell density.

### Induction of Chemical Hypoxia In Vitro

Cobalt chloride (CoCl_2_) is commonly used to stabilize HIF-1α, because it inhibits the HIF-1α degradation, as shown in several *in vitro* settings, including primary human retinal endothelial cells cultures as previously described (Gao et al., 2008; Hu et al., 2012; Li et al., 2017; He et al., 2019). Preliminary studies were carried out and HRECs cultures were treated with various concentrations of CoCl_2_ (100–200 μM), in order to assess cell tolerability for 24 h with MTT test (**Supplementary Data**). The concentration used for all experiments was 200 μM, accordingly to previous CoCl_2_ concentrations tested on retinal ganglion cells (Balaiya et al., 2012; Li et al., 2017). Cells were seeded in Petri dishes (passage number 4, cell density 4×10^5^); after reaching confluence (approximately 80%), cells were treated with CoCl_2_ for 30 min, 2 and 8 h to induce HIF-1α accumulation/nuclear translocation.

### Western Blot

HRECs were cultured in 60 mm Petri dishes (cell density 4×10^5^). Proteins from cell lysates were extracted with RIPA Buffer, including protease and phosphatase inhibitors cocktail (Sigma-Aldrich, St. Louis, MO, USA). Total protein content, in each cell lysate sample, was determined by the BCA Assay Kit (Pierce™ BCA Protein Assay Kit, Invitrogen, Life Technologies, Carlsbad, CA, USA). Extracted proteins (40 µg) were loaded on 4%–12% tris-glycine gel. After electrophoresis proteins were transferred into a nitrocellulose membrane (Invitrogen, Life Technologies, Carlsbad, CA, USA). Immunoblot was preceded by addition of Odyssey Blocking Buffer (LI-COR Lincoln, NE, USA) to membranes. Therefore, membranes were incubated overnight (4°C) with appropriate primary HIF-1α (1:200 dilution) and anti-GAPDH (1:500 dilution) antibodies. GAPDH was selected as control for protein expression, accordingly to previous reports (Botlagunta et al., 2011; Ao et al., 2015; Evrard et al., 2016; Gao et al., 2019). After overnight incubation, the membranes were then incubated with secondary fluorescent antibodies (1: 10,000 dilution) for 1 h at room temperature. Immunoblot was detected through Odyssey imaging system (LI-COR, Lincoln, NE, USA). Densitometry analyses of blots were performed at nonsaturating exposures and analyzed using the ImageJ software (NIH, Bethesda, MD, USA; available at http://rsb.info.nih.gov/ij/index.html). Values were normalized to GAPDH, which was also used as loading control (see **supplemental information** for whole gel membranes immunoblots).

### Extraction of Total RNA and cDNA Synthesis

Extraction of the total RNA was performed with TRIzol Reagent (Invitrogen, Life Technologies, Carlsbad, CA, USA). The A260/A280 ratio of the optical density of RNA samples (measured with Multimode Reader Flash di Varioskan™) was 1.95–2.01. This RNA purity was confirmed with the electrophoresis in nondenaturing 1% agarose gel (in TAE), that showed an adequate RNA purity, concentration, and integrity. cDNA was synthesized from 2 µg RNA with a reverse transcription kit (SuperScript™ II Reverse Transcriptase, Invitrogen, ThermoFisher Scientific, Carlsbad, CA, USA).

### Real-Time Reverse Transcriptase-Polymerase Chain Reaction (qRT-PCR) for PlGF and VEGFA

Real-time RT-PCR was carried out with LightCycler ^®^ 2.0 (Real-Time PCR System Roche Life Science). The amplification reaction mix included iTaq™ Universal SYBR^®^ Green Supermix (Bio-Rad, Hercules, CA, USA) and 1 µl (100 ng) of cDNA. Forty-five amplification cycles were carried out for each sample. Results were analyzed with the 2^-ΔΔ^Ct method. VEGF and PlGF mRNAs expression were normalized to human 18S mRNA levels. Primers used in qPCR for 18S, VEGF-A, PlGF expression are: 18S (human) Forward (5’-AGTCCCTGCCCTTTGTACACA-3’), Reverse (5’-GATCCGAGGGCCTCACTAAAC-3’); PlGF (human) Forward (5′-ATGTTCAGCCCATCCTGTGT-3′) Reverse (5′-CTTCATCTTCTCCCGCAGAG-3′); VEGF-A (human) Forward (5’-GAGGTTTGATCCGCATAATCTG-3’) Reverse (5’-ATCTTCAAGCCATCCTGTGTGC- 3’).

### Analysis of miRNAs

HRECs total RNA, including small RNAs, was obtained following the miRNeasy Mini Kit (21700400, Qiagen), according to the manufacturer’s protocol “Purification of Total RNA, Including Small RNAs, from Animal Cells”. Particularly, for miRNAs isolation, Syn-cel-miR-39-3p miScript miRNA Mimic 5 nM (MSY0000010, Qiagen) was added to each sample before RNA purification in order to monitor miRNAs isolation efficacy. RNA quality and concentration were determined by using NanoDrop 2000c spectrophotometer (Thermo Fisher Scientific, Carlsbad, CA, USA). Gene Amp PCR System 9700 (Applied Biosystems Thermo Fisher Scientific, Carlsbad, CA, USA) was used for reverse-transcription phase. Mature miRNAs were converted in cDNA according the MiScript II Reverse Transcription Kit (218161, Qiagen, Germantown, MD, USA), starting from 615 ng of total RNA.CFX96 Real-Time System C1000 Touch Thermal Cycler (Bio-Rad, Hercules, CA, USA) was used to evaluate the expression levels of hsa-miR-20a-5p (Accession number MIMAT0000075), hsa-miR-20b-5p (Accession number MIMAT0001413); hsa-miR-27a-3p (Accession number MIMAT0000084), hsa-miR-27b-3p (Accession number MIMAT0000419), hsa-miR-206-3p (Accession number MIMAT0000462) and hsa-miR-381-3p (Accession number MIMAT0000736). Real time PCR was carried out with miScript SYBR Green PCR kit (218073, Qiagen, Germantown, MD, USA) and specific miScript primer Assays (MS00003199, MS00003206, MS00003241, MS00031668, MS00003787 and MS00004116, Qiagen, Germantown, MD, USA). The expression of the 6 miRNAs analyzed was normalized by using Ce_miR-39-3p (MIMAT0000010) as control (MS00019789, Qiagen, Germantown, MD, USA).

### TGFβ Pathway qRT-PCR

Total RNA (615 ng) was subjected to reverse-transcription reaction with the Gene Amp PCR System 9700 (Applied Biosystems Life Technologies, Carlsbad, CA, USA) and Quantitect Reverse Transcription kit (205311, Qiagen, Germantown, MD, USA), following the manufacturer’s protocol “Reverse Transcription with Elimination of Genomic DNA for Quantitative, Real-Time PCR”. The expression levels of human TGFB1 (Transforming Growth Factor Beta 1-Gene ID 7040), TGFBR1 (Transforming growth factor beta receptor 1-Gene ID 7046), TGFBR2 (Transforming growth factor beta receptor 2-Gene ID 7048) and SMAD2 (Small mother against decapentaplegic 2-Gene ID 4087) genes were evaluated by real time PCR measurement, by using a CFX96 Real-Time System C1000 Touch Thermal Cycler (BioRad Laboratories, Inc), Quantitect SYBR Green PCR Kit (204143, Qiagen, Germantown, MD, USA) and specific Quantitect Primer Assays (QT00000728, QT00083412, QT00014350 and QT00004207, Qiagen, Germantown, MD, USA) following the manufacturer’s protocol “Two-Step RT-PCR (Standard Protocol)”. Human GAPDH (Gene ID 2597) (QT00079247, Qiagen, Germantown, MD, USA) was used as control to normalize the expression of the 4 genes analyzed; accordingly to previous reports (Botlagunta et al., 2011; Ao et al., 2015; Lin et al., 2015; Rosen et al., 2015; Evrard et al., 2016; Shao and Yao, 2016; Gao et al., 2019; Jiang and Xu, 2019).

### MicroRNA or TGFβ Signaling Pathway Genes Expression Determination Analysis

CFX Manager™ Software (Bio-Rad, Hercules, CA, USA) was used to calculate Cycle threshold (Ct) values. Data analysis was carried out with the 2^-ΔΔ^Ct method. Particularly, ΔCt value for each miRNA or gene profiled was calculated as ΔCt = Ct _miRNA_ – Ct _Ce_miR-39-5p_ or as ΔCt = Ct _gene_ – Ct _GAPDH_. Then, ΔΔCt was calculated as ΔCt_time x_ – ΔCt_time 0_, where time x is the analyzed time point and time 0 is the expression of the target miRNA normalized to Ce-miR-39-5p or of the target gene normalized to GAPDH (Livak and Schmittgen, 2001). Where data are reported as fold-regulation, this was the inverse negative of fold change (2^^-ΔΔ^Ct) for fold change values lower than one (downregulation). In case of upregulation, the fold-regulation was equal to fold change (2^^-ΔΔ^Ct) for fold change values greater than 1.

### Bioinformatics

In order to explore alternative factors and pathways regulated by miRNAs, dysregulated with induction of chemical hypoxia in human retinal endothelial cells, we predicted the combinatorial effect of hsa-miR-20a-5p, hsa-miR-20b-5p, hsa-miR-27a-3p, has-miR-27b-3p, has-miR-260b-3p, and has-miR-381-3p on biological pathways by means of the DIANA miRPath webserver (Vlachos et al., 2015). The miRNA:target interactions were analyzed with application of Tarbase algorithm (Riffo-Campos et al., 2016), which is based on experimental validated miRNA:target interaction.

### Statistical Analysis

All results were reported as mean ± SD from four independent in-vitro experiments, where each group was triplicated in plates as technical replicate. The results were analyzed using one-way ANOVA, followed by Tukey-Kramer post-hoc multiple comparisons test. Differences between groups were considered significant for p-value < 0.05. Graphs design and statistical analysis were carried out with GraphPad Prism 5 software (GraphPad Inc., San Diego, CA, USA).

## Results

### Chemical Hypoxia in HRECs and Angiogenic Factors

**Figure 1:**
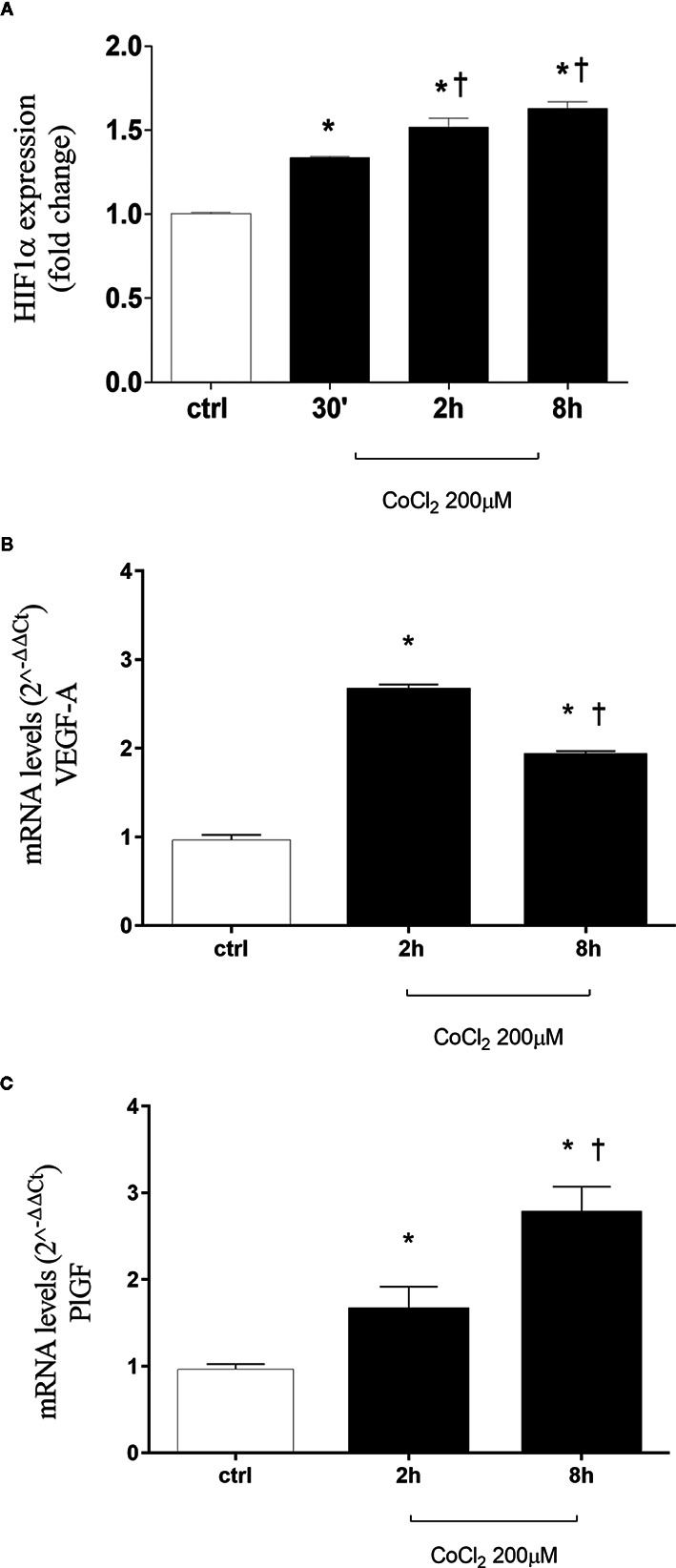
CoCl_2_ treatment induces HIF-1α stabilization, vascular endothelial growth factor (VEGFA), and placental growth factor (PlGF) expression in human retinal endothelial cells. **(A)** Densitometric analysis of western blot of HIF-1α and GAPDH in human retinal endothelial cells (HRECs) exposed to CoCl_2_ for 30 min, 2 h and 8 h; each bar represents the mean value ± SD (n=4). *p< 0.05 vs. control; ^†^p < 0.05 vs. 30 min CoCl_2_ treatments. **(B)** CoCl_2_ treatment increased VEGF-A mRNA expression. Each bar represents the mean value ± SD. *p < 0.05 CoCl_2_ vs. control; ^†^p < 0.05 8 h vs. 2 h CoCl_2_ treatment; (n=4). **(C)** CoCl_2_ treatment increased PlGF mRNA expression. Each bar represents the mean value ± SD. *p < 0.05 CoCl_2_ vs. control; ^†^p < 0.05 8 h vs. 2 h CoCl_2_ treatment; (n=4). The mRNA levels were evaluated by qRT-PCR.

CoCl_2_ treatment, by inhibition of HIF-1α degradation, significantly increased stabilization of HIF-1α protein in HRECs (**Figure 1A** and **Supplementary Data**). HIF-1α is a well-known inducer of VEGFA and PlGF (Aiello et al., 1994; Ozaki et al., 1999; Arjamaa and Nikinmaa, 2006; Abu El-Asrar et al., 2012; Zimna and Kurpisz, 2015; Charnock-Jones, 2016; Rodrigues et al., 2016; Mitsui et al., 2018), but the HIF-1α protein levels did not correlate with expression pattern of VEGFA, within the analyzed time-points (**Figure 1B**). Two hours after CoCl_2_ treatment, VEGFA expression increased, compared to control HRECs. While, after 8 h, VEGFA levels significantly (p<0.05) decreased, compared to levels detected 2 h after, CoCl_2_ treatment. On the other hand, the expression pattern of PlGF correlated with HIF-1α protein levels, within the analyzed time-points (**Figure 1C**).

### Expression Analysis of miRNAs Induced by CoCl_2_ Treatmet of HRECs

**Figure 2:**
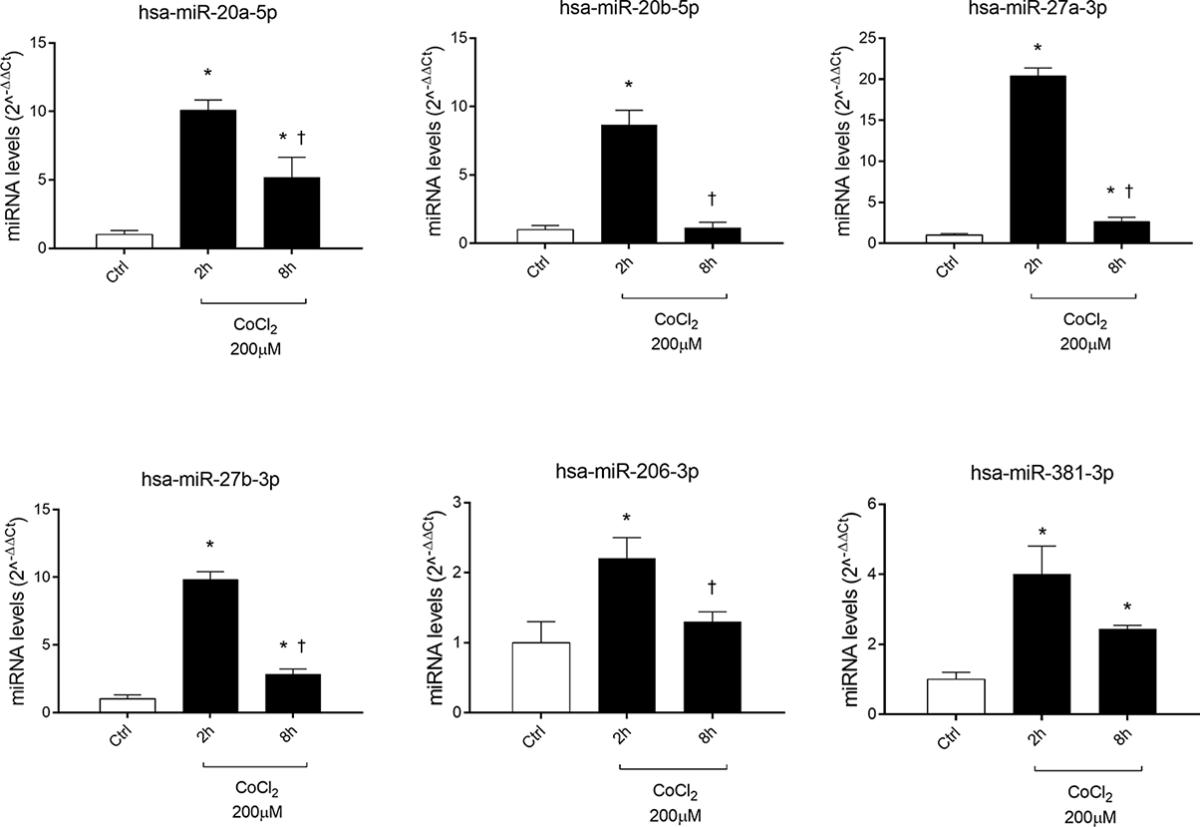
Pattern expression of microRNAs (miRNAs) in human retinal endothelial cells (HRECs) treated with CoCl_2_, for 2 and 8 h treatment. Expression of miRNAs was analyzed with qRT-PCR. Each bar represents the mean value ± SD. *p < 0.05 200 µM CoCl_2_ vs. control (ctrl); ^†^p<0.05 8h vs. 2h CoCl_2_ treatment; (n=4).

**Figure 3:**
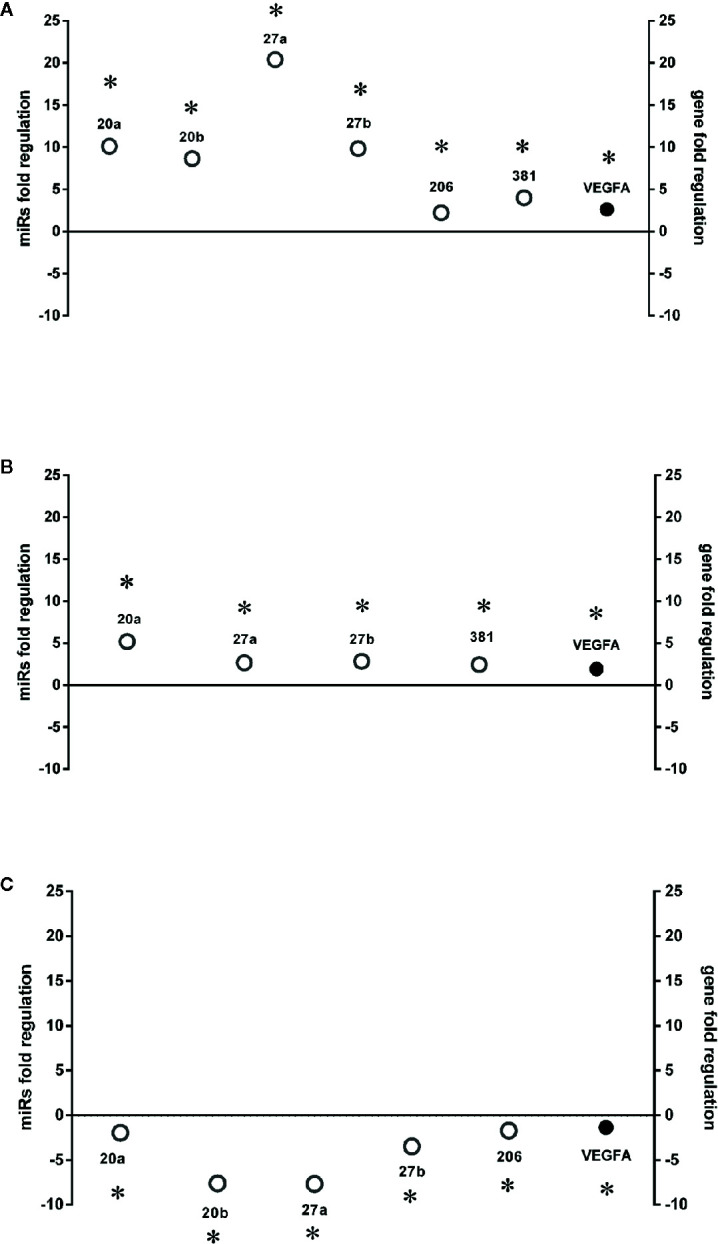
HIF-1α stabilization induced microRNAs (miRNAs) and correlation with vascular endothelial growth factor (VEGFA) expression. Correlation of microRNAs and VEGFA expression (Fold regulation). **(A)** *p < 0.05 2 h CoCl_2_ treatment vs. control; **(B)** *p < 0.05 8 h CoCl_2_ vs. control (ctrl); **(C)** *p < 0.05 8h vs. 2h CoCl_2_ treatment; (n=4).

Six miRNAs (miR-20a-5p, miR-20b-5p, miR-27a-3p, miR-27b-3p, miR-260b-3p, miR-381-3p), out of eight analyzed, were found to be significantly (p<0.05) dysregulated in HRECs, treated with 200 µM CoCl_2_, compared to control cells (**Figure 2**). All dysregulated miRNAs were found to be significantly (p<0.05) upregulated, 2 h after CoCl_2_ treatment, compared to control cells (**Figure 3A**). On the contrary, four miRNAs were significantly (p<0.05) dysregulated (upregulated) 8 h after CoCl_2_ treatment, compared to control cells (**Figure 3B**). Furthermore, after 8 h of exposure to CoCl_2_, five miRNAs (miR-20a, miR-20b, miR-27a, miR-27b, miR-206-3p) were significantly downregulated (p<0.05), compared to levels detected in cells treated for 2 h with 200 µM CoCl_2_, with exception of miR-381-3p (**Figure 3C**).

### TGFβ Signaling Pathway in HRECs Challenged With CoCl_2_

**Figure 4:**
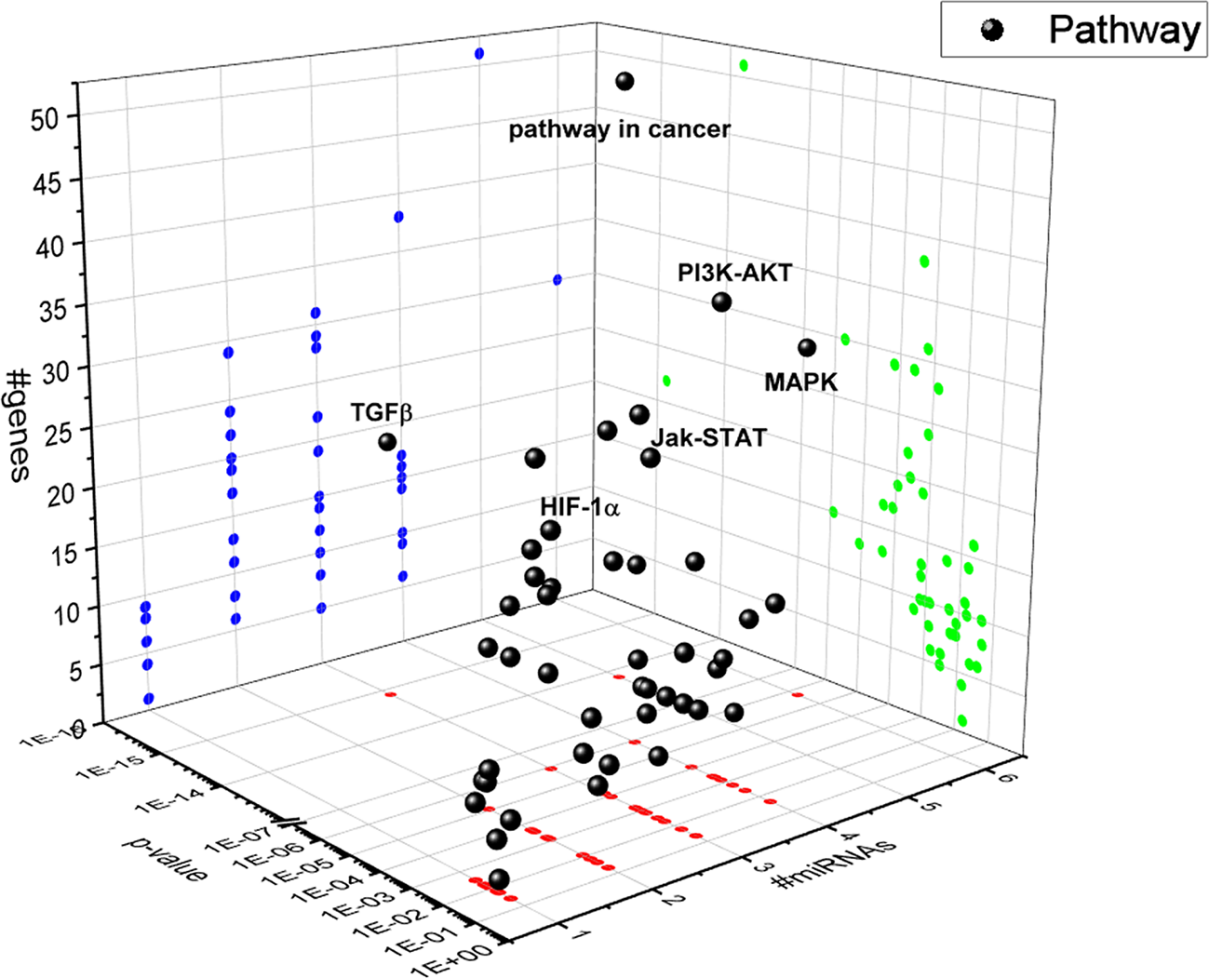
3D scatter plot of pathways regulated by HypoxamiRs in human retinal endothelial cells (HRECs), challenged with CoCl_2_. Blue dots represent #gene projection of #miRNAs dimension. Green dots represent p-value projection of #genes dimension. Red dots represent #miRNA projection of p-value dimension.

A bioinformatic approach was used to predict the combinatorial effect of miR-20a-5p, miR-20b-5p, miR-27a-3p, miR-27b-3p, miR-206-3p, and miR-381-3p on biological pathways. The pathways dysregulated by these miRNAs were predicted by means of DIANA miRPath, applying the Tarbase algorithm, which generates, as output, pathways related to experimental validated miRNA:mRNA interactions (Vlachos et al., 2015). Based on this bioinformatic approach, we found that the TGFβ signaling pathway was the top-scored among the pathways significantly (p<0.05) dysregulated by hypoxia-induced miRNA in HRECs (**Figure 4**). The HIF-1α pathway was predicted to be regulated by miR-20a-5p, miR-20b-5p, miR-27a-3p, miR-27b-3p, miR-260-3p, miR-381-3p, according to the *in vitro* model of retinal chemical hypoxia. Moreover, PI3K-AKT, MAP kinases and Jak-STAT signaling pathways were predicted to be modulated by the six miRNAs, that were dysregulated in HRECs treated with CoCl_2_.

**Figure 5:**
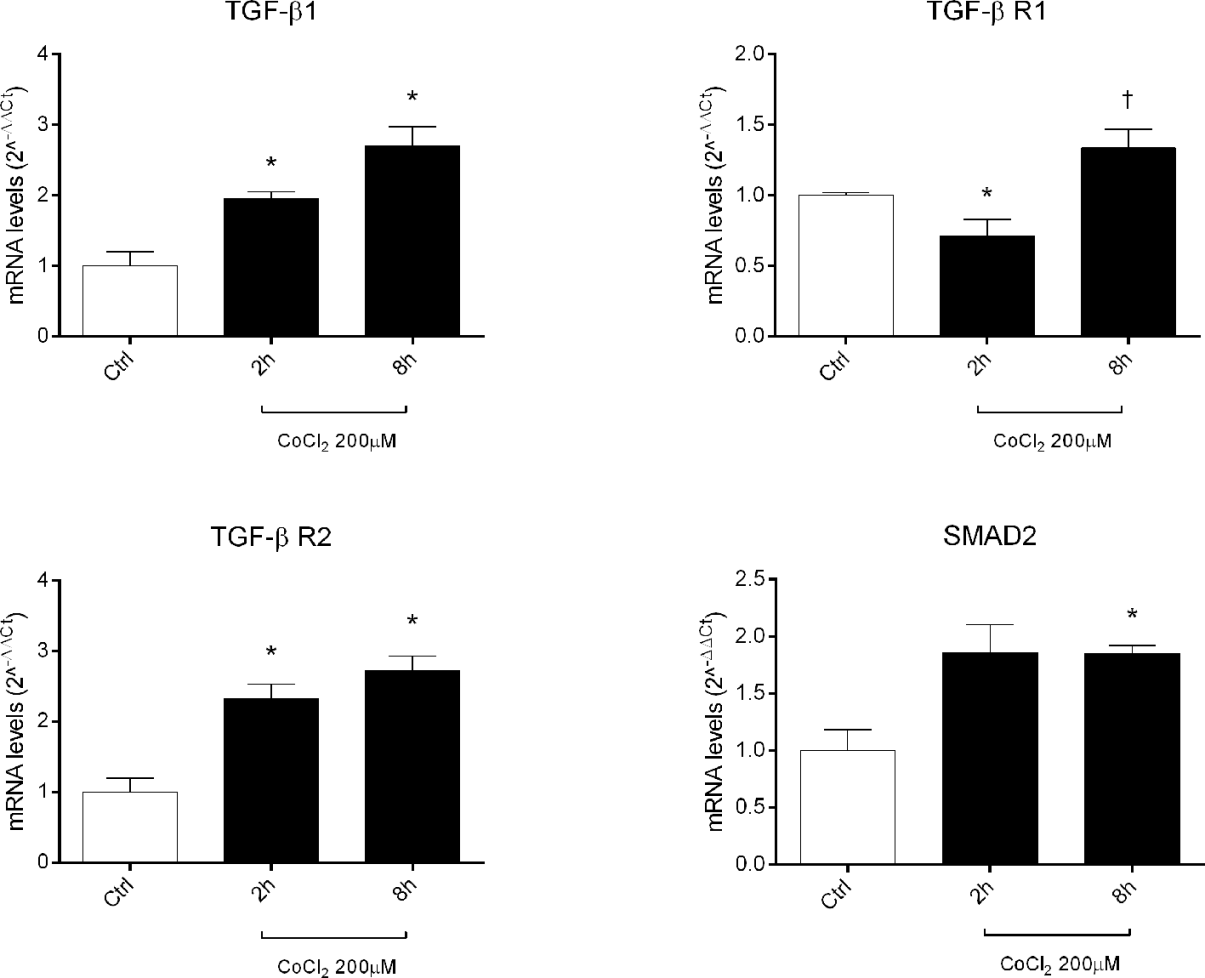
Expression of genes of TGFβ signaling pathway in human retinal endothelial cells (HRECs) treated with CoCl_2_, for 2 and 8 h. The mRNA levels were evaluated by qRT-PCR. Each bar represents the mean value ± SD. *p < 0.05 CoCl_2_ vs. control (ctrl); ^†^p < 0.05 8h vs. 2h CoCl_2_ treatment. (n=4).

**Figure 6:**
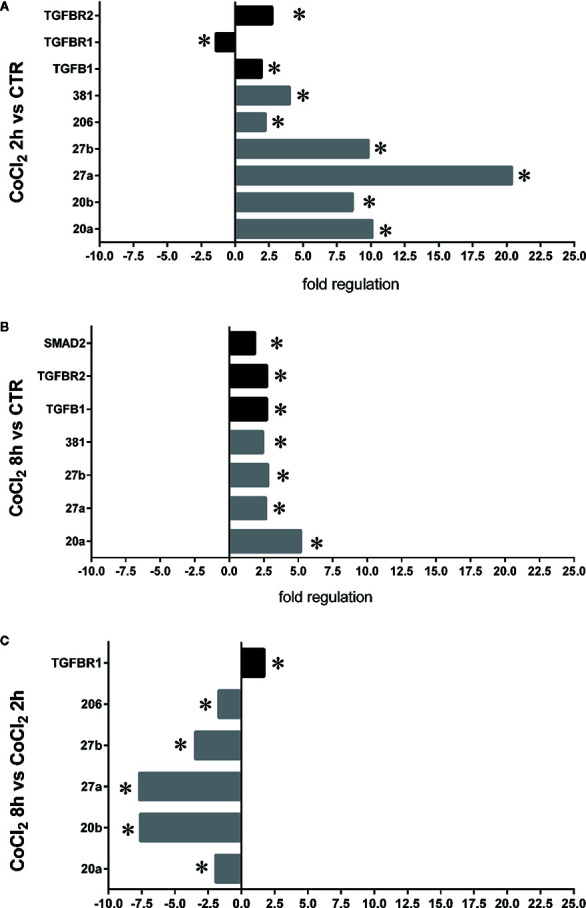
Correlation of microRNAs (miRNAs) and TGFβ signaling pathway genes expression in human retinal endothelial cells (HRECs), under chemical hypoxic stimuli. Fold regulation of miRNAs and TGFβ signaling genes: **(A)** *p < 0.05 2 h CoCl2 treatment vs. control (ctr); **(B)** *p < 0.05 8 h CoCl2 treatment vs. control (ctr); **(C)** *p < 0.05 8 h vs. 2 h CoCl_2_ treatment; (n=4).

Therefore, we focused our study on analysis of transcription of TGFβ signaling pathway genes (*TGFB1* encoding for TGFβ1, *TGFBR1* encoding for the TGFβR1 receptor, *TGFBR2* encoding for the TGFβR2 receptor and *SMAD2* encoding for SMAD2), in HRECs treated with 200µM CoCl_2_ (**Table 1**). These genes were significantly (p<0.05) dysregulated in HRECs, 2 and 8 h after CoCl_2_ treatment (**Figure 5**). Furthermore, we correlated gene expression with dysregulated miRNAs in the analyzed time-points (**Figure 6**, **Table 1**). After 2 h of CoCl_2_ treatment, *TGFBR2* and *TGFB1* gene expression increased significantly, TGFBR1 decreased (p<0.05) (**Figure 6A**), and all analyzed miRNA were significantly upregulated, particularly miR-27a. The mRNA of *TGFBR1* is an experimental validated target of miR-20a, miR-20b, miR-27a, miR-27b, and miR-381, therefore the upregulation of this miRNAs significantly decreased the *TGFBR1* mRNA levels (**Table 1**). Moreover *TGFB1*, and *TGFBR2* are experimental validated targets of miR-27a and miR-20a, respectively, which even if overexpressed did not reduce the expression of these two genes, 2 h after CoCl_2_ treatment (**Figure 6A**). Eight hours after CoCl_2_ treatment, miR-20a, miR-27a, miR-27b, and miR-381-3p were significantly (p<0.05) upregulated in HRECs, compared to control cells (**Figure 6B**). This pattern of miRNA expression positively correlated with *TGFB1*, *TGFBR2*, and *SMAD2* expression (**Figure 6B**). Although not significantly, 8h after CoCl_2_ treatment, mRNA levels of TGFβ1, TGFβR2 and SMAD2 were higher, compared to HRECs treated for 2 h with CoCl_2_ (**Table 1**). On the other hand, TGFβR1 mRNA expression levels were significantly upregulated 8 h after CoCl_2_ treatment, compared to cells treated for 2 h with CoCl_2_ (**Figure 6C**). This expression pattern negatively correlated with downregulation of miR-20a, miR-20b, miR-27a, miR-27b, and miR-206, according to the opposite trend observed 2 h after chemical hypoxia.

**Table 1:** Differential expression of genes of the TGFβ signaling pathway.

Gene	Fold regulationCoCl_2_ 2h vs CTR(p value)	Fold regulationCoCl_2_ 8h vs CTR(p value)	Fold regulationCoCl_2_8h vs 2h(p value)	Regulating miRNAs (tarbase)
TGFB1	1.9466 (p<0.01)	2.7128 (p<0.05)	1.3936	miR-27a
TGFBR1	−1.419 (p<0.05)	1.3352	1.7166 (p<0.05)	miR-20a, miR-27a, miR-27b, miR-20b, (microT-CDS), miR-381 (microT-CDS)
TGFBR2	2.327 (p<0.05)	2.7213 (p<0.05)	1.1694	miR-20a, miR-20b
SMAD2	1.5014	1.8547 (p<0.05)	1.2354	miR-20b, miR-206 (microT-CDS), miR-381 (microT-CDS)

The microRNAs (miRNAs), targeting each gene, were predicted with application of Tarbase, or whenever written with microT-CDS algorithm.

## Discussion

Previous data report that eight miRNAs (miR-20a-5p, miR-20a3p, miR-20b-5p, miR-106a-5p, miR-27a-5p, miR-27b-3p, miR-206-3p, and miR-381-3p) were significantly dysregulated both in serum and retina of 5–10 months diabetic mice (Platania et al., 2019). Because retinal hypoxia is detrimental in DR, exacerbating retinal damage and angiogenesis (Aiello et al., 1994; Arjamaa and Nikinmaa, 2006; Abu El-Asrar et al., 2012), we aimed at testing the hypothesis that these miRNAs would be modulated in human retinal endothelial cells, treated with CoCl_2_ in order to stabilize HIF-1α.

In DR, the role of angiogenesis linked to hypoxic events (i.e. increased VEGFA production stimulated by HIF-1α) has been largely proven (Arjamaa and Nikinmaa, 2006; B. Arden and Sivaprasad, 2012; Kurihara et al., 2014; Li et al., 2017). Furthermore, HIF-1α can induce expression of another proangiogenic factor, the PlGF (Tudisco et al., 2014; Lazzara et al., 2019). In this study we found a correlation, in terms of time-dependent expression, between HIF-1α and PlGF, after CoCl_2_ treatment (**Figure 1**). Instead, VEGF mRNA levels did not correlate with HIF-1α protein (**Figure 1**). For this reason, we hypothesized that other factors could regulate VEGFA expression in an *in vitro* model of chemical hypoxia, such as miRNAs. Involvement of miRNAs in retinal neovascular diseases has been widely studied (Romano et al., 2017; Natoli and Fernando, 2018; Martinez and Peplow, 2019; Platania et al., 2019). We found that six miRNAs (miR-20a-5p, miR-20b-5p, miR-27a-3p, miR-27b-3p, miR-206-3p, miR-381-3p), out of eight tested, were dysregulated in human retinal endothelial cells after CoCl_2_ treatment (**Figure 2**). These miRNAs have been previously found to be either HypoxamiRs (bearing HREs in their promoting region) or linked to hypoxic microenvironment (Nallamshetty et al., 2013; Yue et al., 2013; Choudhry and Mole, 2016; Gupta et al., 2018; Lu et al., 2018).

After 8 h, similarly to VEGFA expression, we found a shift in expression pattern of miRNAs, compared levels detected 2 h after CoCl_2_ treatment (**Figure 3**). Experimental validated miRNA : VEGFA mRNA interactions were found for miR-20a-5p and miR-20b-5p (Platania et al., 2019), and in hepatocellular carcinoma for miR-381-3p (Tsai et al., 2017; Wang et al., 2018). Therefore, VEGFA expression levels could be related to the expression pattern of miRNAs, 2 to 8 h after stabilization of HIF-1α, because VEGFA is a target of miR-20a, miR-20b, miR-381, and indirectly of miR-27b (Veliceasa et al., 2015). On the contrary, PlGF is not a validated or predicted target of any miRNAs dysregulated in HRECs treated with CoCl_2_. Particularly, the role of PlGF in regulation retinal angiogenesis, under hypoxic stimuli, is still unknown. On the other hand, several reports support the detrimental role of PlGF in the pathogenesis and progression of DR (Carmeliet et al., 2001; Huang et al., 2015), likely through HIF-1α, or indirectly by miRNAs and the PI3K/AKT signaling pathways (**Figure 4**) (Zhou et al., 2016; Jin et al., 2018).

Therefore, our hypothesis is based on retinal angiogenesis regulated by miRNAs under hypoxic stimuli, and miRNAs can be considered alternative and/or ancillary components to VEGFA and PlGF pathways. Indeed, we analyzed other putative miRNAs targets (gene and pathways) and identified, through a bioinformatic approach, the TGFβ signaling pathway as the top-scored pathway dysregulated by identified miRNAs (**Figure 4**). Then, we found that miRNAs, dysregulated after CoCl_2_ treatment, (miR-20a-5p, miR-20b-5p, miR-27a-3p, miR-27b-3p, miR-206-3p, miR-381-3p) influenced mRNA levels of TGFβ1, TGFβR1, TGFβR2 and SMAD2, according to experimental validated miRNA:mRNA interactions (**Figures 5** and **6**, **Table 1**). TGFβ1, TGFβR2 and SMAD2, were upregulated 2 and 8 h after HIF-1α stabilization. Interestingly, the expression of TGFβR1 receptor, which is target of most of analyzed miRNAs (**Table 1**), correlated with expression pattern shift of miRNAs at 2 h and 8 h after CoCl_2_ treatment. Several reports support a detrimental role of TGFβR1 in DR, particularly, TGFβR1 immunoreactivity was found to be increased in retinal capillaries of diabetic rats (Gerhardinger et al., 2009; van Geest et al., 2010).

The HIF-1/TGF-β1 axis, and related stimulation of angiogenesis, has been investigated in different experimental settings (Han et al., 2013; Mingyuan et al., 2018), including endothelial cells (Iruela-Arispe and Sage, 1993; Peshavariya et al., 2014). On the contrary, few reports demonstrated a putative link between HIF-1α/miRNAs/TGFβ signaling pathway and angiogenesis (Xing et al., 2014). Furthermore, only one study analyzed the role miRNAs in regulation of hypoxia-TGFβ-angiogenesis pathway in a model of corneal neovascularization (Zhang Y. et al., 2019). According to our findings, miR-27 was reported to be involved in regulation of HIF-1/TGFβ axis, at least in an *in vitro* model of cardiac ischemia (Zhang X. L. et al., 2019). However, there are still no evidences about a putative link in retinal disease between hypoxia, miRNAs, VEGFA, and TGFβ pathway.

**Figure 7:**
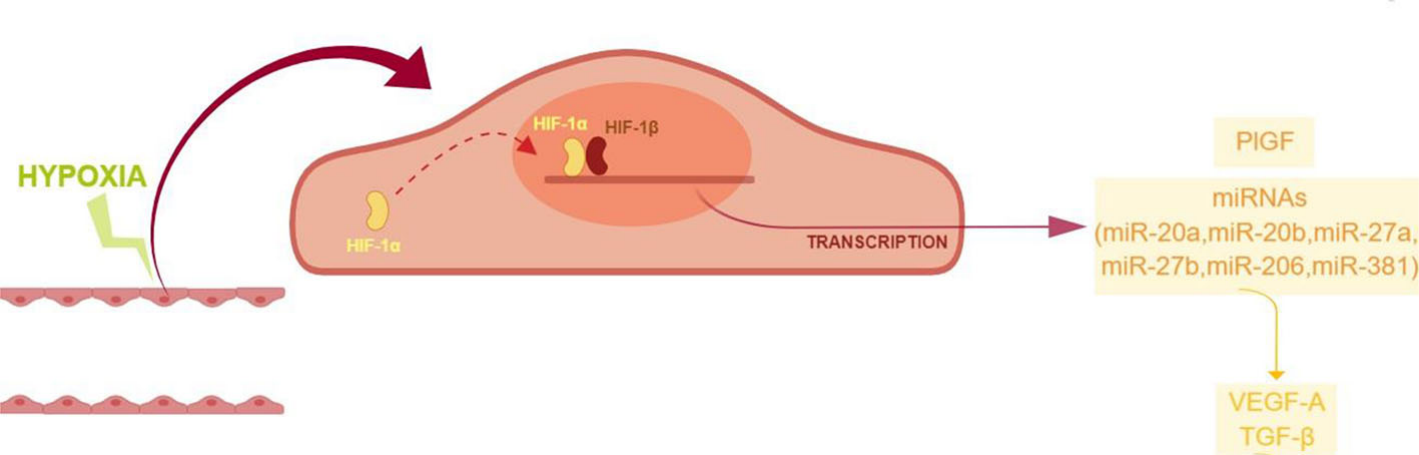
Proposed model of angiogenic shift in retinal endothelial cells exposed to chemical hypoxia. CoCl_2_-induced hypoxia leads to the stabilization of HIF-1α, with the subsequent translocation into the nucleus and transcription of hypoxia-related genes.

High throughput miRNA expression analysis on retinal endothelial cells, challenged with chemical hypoxic stimuli, could reveal the involvement of other miRNAs, along with the focused set analyzed in this study. However, those high throughput analyses are expensive and need quantitative qPCR validation (de Ronde et al., 2018). Despite the small set of analyzed miRNAs, our study suggested that ocular neovascularization, during hypoxia, would be promoted by the upregulation of PlGF and other factors induced by HIF-1α/miRNAs, i.e. VEGFA, and genes of the TGFβ1 signaling pathway (**Figure 7**). Therefore, these data warranting further *in vivo* studies to explore the use of pharmacological/molecular approach such as antagomiRs and agomir.

Indeed, the present findings highlighted that proangiogenic factors are worthy to be further explored as potential targets for pharmacological modulation of local retinal hypoxic events, which are generally transient but detrimental in retinal degenerations.

## Data Availability Statement

The datasets generated for this study are available on request to the corresponding author.

## Author Contributions 

CB, MD’M, and SR made substantial contributions to conception, design, and interpretation of data. FL, MT, and CP carried out experiments. FL, MT, CP, FP, and CG carried out formal analysis of data. FL, MT, CP, and CB wrote initial draft of the manuscript. CB, MD’M, SR, FD, and MG reviewed the manuscript critically for important intellectual content and gave final approval of the version to be submitted.

## Conflict of Interest

The authors declare that the research was conducted in the absence of any commercial or financial relationships that could be construed as a potential conflict of interest.
