# Supplementary material for: Stabilization of HIF-1α in Human Retinal Endothelial Cells Modulates Expression of miRNAs and Proangiogenic Growth Factors
Source: Front Pharmacol. 2020 Jul 17;11:1063. doi: 10.3389/fphar.2020.01063 (PMC7396674; doi:10.3389/fphar.2020.01063)
Supplement: Supplementary file 1 [file DataSheet_1.docx]

**SUPPLEMENTAL: Stabilization of HIF-1α in human retinal endothelial cells modulates expression of miRNAs and pro-angiogenic growth factors**

**Francesca Lazzara^1^*, Maria Consiglia Trotta^2^*, Chiara Bianca Maria Platania^1^*, Michele D’Amico^2^, Francesco Petrillo^2^, Marilena Galdiero^2^, Carlo Gesualdo^4^, Settimio Rossi^4^, Filippo Drago^1,3^ and Claudio Bucolo^1,3#^.**

^1^Department of Biomedical and Biotechnological Sciences, School of Medicine, University of Catania, Catania, Italy; ^2^Department of Experimental Medicine, Division of Pharmacology, University of Campania “Luigi Vanvitelli”, Naples, Italy; ^3^Center for Research in Ocular Pharmacology-CERFO, University of Catania, Catania, Italy; ^4^Eye Clinic, Multidisciplinary Department of Medical, Surgical and Dental Sciences, University of Campania “Luigi Vanvitelli”, Naples, Italy.

*these authors have contributed equally to the work

# corresponding author: Prof. Claudio Bucolo. Via Santa Sofia 97. 95125 Catania, Italy. Telephone: +390954781196. E-mail: [claudio.bucolo@unict.it](mailto:claudio.bucolo@unict.it)

**Figure 1S Hif-1α westernblot**

**
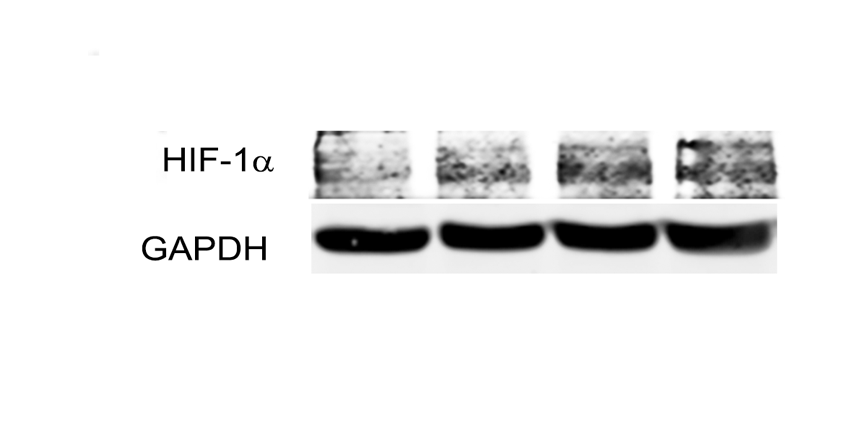
**

**Figure 2S Ponceau of membrane after gel transfer**


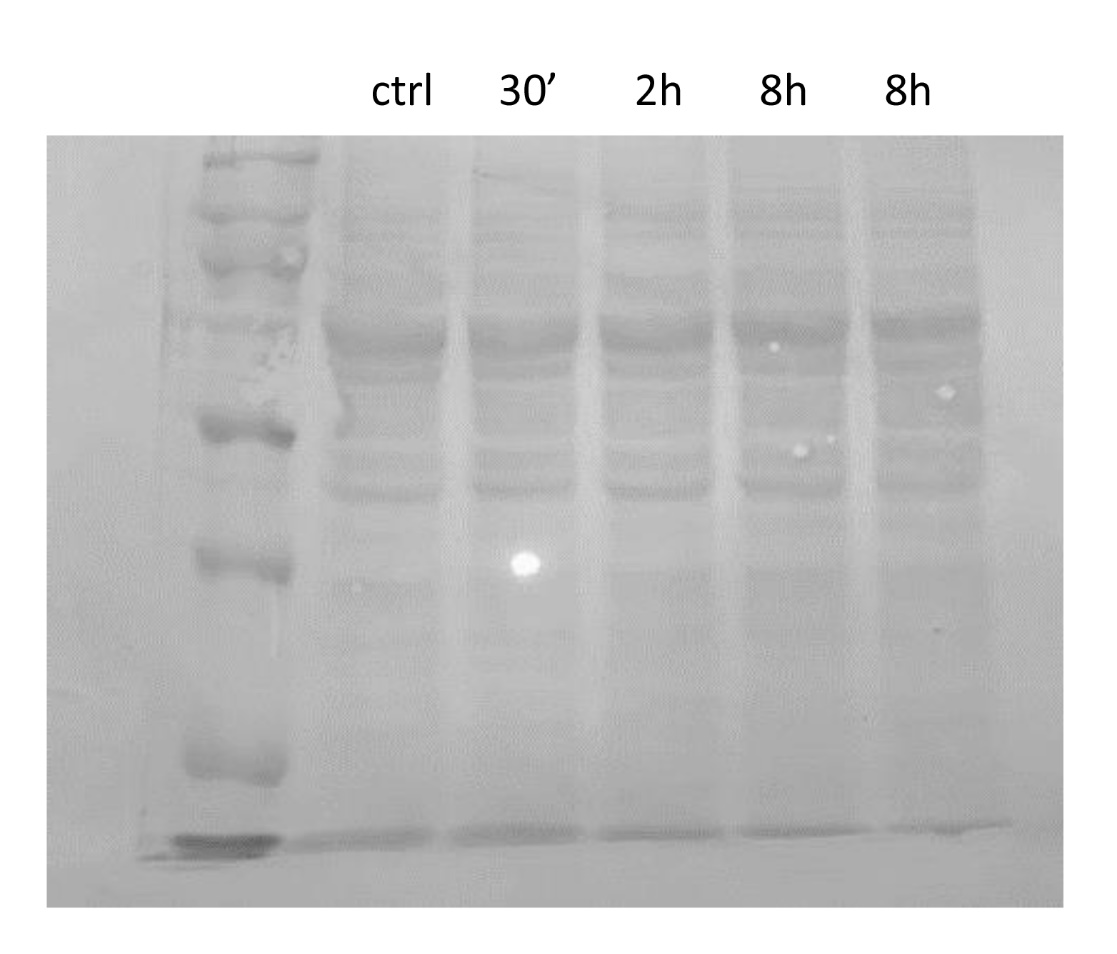


**Figure 3S Whole membranes after immunoblotting for HIF1α and GAPDH**


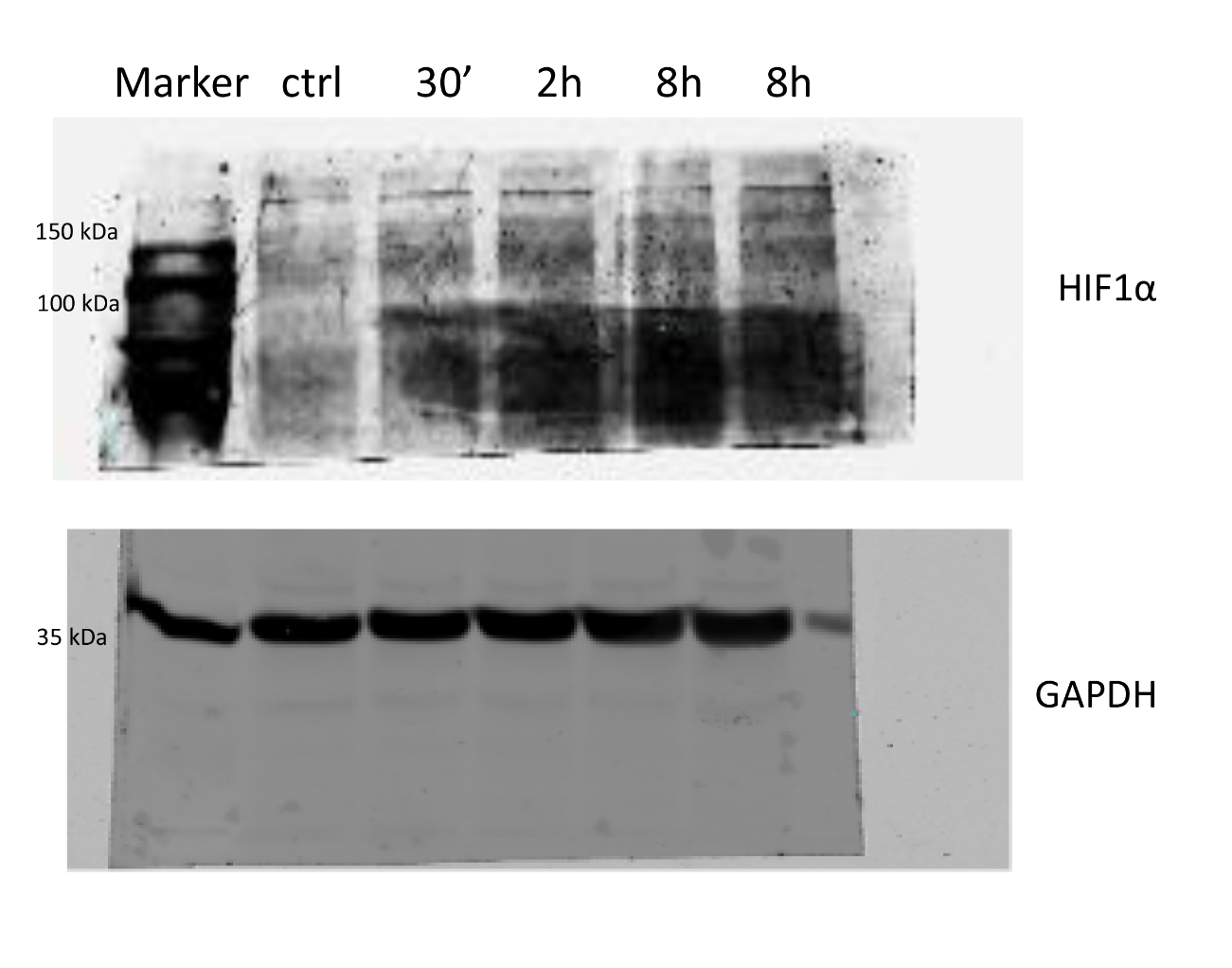


**Figure 4S: MTT assay. Effects of CoCl_2_ (100-200 μM for 6 and 24 h) on primary microglia HRECs. Bars are mean ± SD of at least three independent experiments.**





**Table 1S: Ct values for GAPDH (used as control for TGFβ signaling pathway) and Cel-miR-39-3p (used as control for miRNAs) obtained from qRT-PCR analysis.** Ct values for GAPDH and Cel-miR-39-3p were not significantly modified in HRECs exposed to CoCl_2_ (200 μM) for 2 or 8 hours, in comparison to control cells. Data are reported as mean ± SD of four independent experiments.

|  | **CTRL** | **CoCl2 2h** | **CoCl2 8h** |
| --- | --- | --- | --- |
| **GAPDH** | 22,61 ± 0,3 | 22,44 ± 0,4 | 22,31 ± 0,6 |
| **Cel-miR-39-3p** | 19,73 ± 0,5 | 19,91 ± 0,6 | 19,80 ± 0,3 |
