# Supplementary figures and images for: Stabilization of HIF-1α in Human Retinal Endothelial Cells Modulates Expression of miRNAs and Proangiogenic Growth Factors
Source: Front Pharmacol. 2020 Jul 17;11:1063. doi: 10.3389/fphar.2020.01063 (PMC7396674; doi:10.3389/fphar.2020.01063)

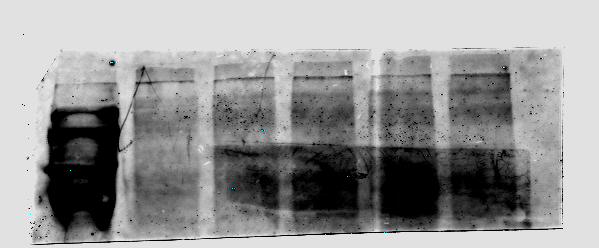

Supplement: Supplementary file 3 [file Image_2.tif]
